# The Comparison of Outcomes of Transvaginal Mesh Surgery with and without Midline Fascial Plication for the Treatment of Anterior Vaginal Prolapse: A Randomized Controlled Trial

**DOI:** 10.3390/jcm10091888

**Published:** 2021-04-27

**Authors:** Ching-Hsiang Chiang, Chun-Shuo Hsu, Dah-Ching Ding

**Affiliations:** 1 Department of Urology, Hualien Tzu Chi Hospital, Buddhist Tzu Chi Medical Foundation, Hualien 970, Taiwan; Jimjim4562001@gmail.com; 2 School of Medicine, Tzu Chi University, Hualien 970, Taiwan; 3 Department of Obstetrics and Gynecology, Dalin Tzu Chi Hospital, Buddhist Tzu Chi Medical Foundation, Chayi 622, Taiwan; 4 Department of Obstetrics and Gynecology, Hualien Tzu Chi Hospital, Buddhist Tzu Chi Medical Foundation, No. 707, Chung-Yang Rd., Sec. 3, Hualien 970, Taiwan; 5 Institute of Medical Sciences, College of Medicine, Tzu Chi University, Hualien 970, Taiwan

**Keywords:** mesh, pelvic organ prolapse, plication, life quality

## Abstract

The aim of this study was to compare the clinical outcomes of transvaginal mesh (TVM) surgery with and without midline fascial plication for anterior prolapse repair. This is a prospective randomized trial in a teaching hospital. This study compared patients with anterior vaginal wall prolapse (POP-Q Ba > −1) who were randomly assigned to either transvaginal mesh (TVM, Avaulta Solo^TM^, CR Bard. Inc., Covington, GA, USA polypropylene mesh delivery system) (group A, n = 32) or TVM with concomitant midline fascial plication (group B, n = 32). The outcomes of anatomy correction and life quality were evaluated using a pelvic organ prolapse quantification system and questionnaires. Sixty-four patients were included from January 2011 through April 2014 in this study. Group A had a mean age of 63.7 years and a body mass index (BMI) of 25.4 kg/m^2^. Group B had a mean age of 62.9 years and a BMI of 25.4. The mean follow-up duration was 18.6 months (range 12–50). At the 12-month follow-up, anatomic recurrence was higher in Group A (5/31, 16.1%) than in Group B (1/30, 3.3%) but without statistical significance (*p* = 0.19). Improvements in symptoms and quality of life were not significantly different between the two groups. Mesh extrusion was detected in three of 61 patients (4.9%): two from group A (6.7%) and one from Group B (3.2%). TVM with concomitant midline fascia repair for anterior vaginal prolapse had a comparable anterior support and mesh exposure rate compared with TVM alone. Trial Registration: IRB-B09904021

## 1. Introduction

Pelvic organ prolapse (POP) is an anatomical change wherein the female pelvic organs (vagina, bladder, uterus, and/or rectum) descend into or through the vagina. It is a common problem, with 50% of women reporting symptoms of pelvic organ prolapse in the UK [1]. The lifetime risk of surgery for POP reaches up to 19%, with more than 300,000 prolapse surgeries performed in the US annually [2,3]. It directly impacts bladder and bowel function, affecting the social, psychological, occupational, domestic, and sexual aspects of the patient’s life [4]. The etiology of POP is multifactorial, including obstetric risk factors [5,6] (vaginal childbirth, advanced age, increased body mass index, constipation, smoking, and prior hysterectomy) and genetic influences such as connective tissue disorders [7,8,9,10,11].

Anterior vaginal wall prolapse is the most common POP subtype. It is two and three times more common than posterior and apical vaginal prolapse, respectively [12,13]. In surgical management, the goal of anterior segment reconstruction is to restore normal pelvic anatomy and to regain normal bladder and sexual functions. Anterior colporrhaphy with native tissue repair has been the standard procedure for managing anterior compartment prolapse and has an acceptable success rate of 80–100% in case series [14]. However, it only achieves a 40–60% success rate in randomized trials and has a high recurrence rate of 38% within one to three years of follow-up [14,15]. Transvaginal commercial mesh devices have been introduced to improve the effectiveness of anterior POP repairs. Despite the better success rate, it causes a longer operating time, greater blood loss, and mesh exposure at a rate of 10.4% [15,16]. In combination procedures, Karp et al. suggest that the addition of concomitant midline fascial plication in anterior vaginal prolapse repair with a non-crosslinked biologic graft vaginal prolapse enhances the anatomic outcome [17]. Concomitant midline fascia plication is beneficial, but the data on synthetic mesh overlying is limited.

We hypothesize that the outcome of transvaginal mesh (TVM) plus fascia plication is better than TVM alone. The study aimed to compare the subjective and objective outcomes between transvaginal mesh placement alone and a combined procedure (transvaginal permanent mesh repair with concomitant midline fascia plication) to treat anterior compartment prolapse during a 12-month follow-up.

## 2. Methods

### 2.1. Patients

The pelvic organ prolapse staging was quantified using the POP-Q system [18]. POP-Q stage II or above patients, who were 50–75 years old, were eligible.

Exclusion criteria were as follows: diagnosis of coagulation disorder, renal failure, cognitive impairment, pelvic radiation history, history of implants for pelvic organ prolapse, and previous vaginal surgery.

History taking and physical examination were performed for each patient. This study was conducted in the Dalin Tzu Chi Hospital between January 2011 and April 2014. The protocol was approved by the Research Ethic Committees of Dalin Tzu Chi Hospital (IRB-B09904021). Each participant signed the informed consent before the procedure.

### 2.2. Trial Design and Interventions

This randomized, nonblind, phase 3 trial was conducted in the Dalin Tzu Chi Hospital. Randomization was performed with envelope-generated randomization at the time of the procedure. Patients were assigned randomly, in a 1:1 ratio, to receive TVM with Avaulta Solo^TM^ polypropylene mesh delivery system (CR Bard Inc., Covington, GA, USA) or TVM with concomitant midline fascial plication.

### 2.3. Surgical Technique

Each patient received a single dose of intravenous cefazolin (1 g) for antibiotics prophylaxis at the time of anesthesia induction.

Regarding the Avaulta solo^TM^ procedure [19], patients were positioned in a lithotomy position and received general anesthesia. A dilated epinephrine solution (1:1000) was injected beneath the vaginal mucosa layer. Then, the full length of the vaginal wall was opened and dissected to develop the vesicovaginal to pubic ramus or rectovaginal space to ischial spine. Two small incisions were made in the bilateral groin region. Then, an Avulta trocar was used to extend the mesh through bilateral groin incisions. Two arms of mesh were then passed through the incisions following the trocar guide. After mesh placement, anterior colporrhaphy was then performed. Cystoscopy was used to check the integrity of the bladder. Then, the position of the mesh was adjusted to relieve tension.

Regarding the midline plication procedure [20], the vaginal wall was incised vertically, and the muscularis (pubocervical fascia) was dissected from the vaginal epithelium. Then, the vaginal muscularis was plicated with 2-0 vicryl using an interrupted manner. After plication, we removed redundant vaginal epithelium and approximated with a 2-0 vicryl running suture.

To conserve or remove the uterus was a patient choice.

### 2.4. Outcomes

The primary outcome was an anatomical failure rate at 12 months after operation. Outcomes were divided into objective and subjective outcomes. Objective outcomes were evaluated using the pelvic organ prolapse quantification system. Subjective outcomes were evaluated with questionnaires. The Pelvic Floor Distress Inventory Short form 20 (PFDI- 20) and Pelvic Impact Questionnaire Short Form 7 (PFIQ-7) were administered at 12 months preoperatively and postoperatively. Anatomic failure was defined as the most dependent portion reaching stage 2 (patient with any POP-Q point 0) or a reported vaginal bulge on the PFDI-20 questionnaire.

The secondary outcome was the rate of mesh exposure, which was defined as the patient being found to have any visible or palpable mesh material during physical examination after operation [21].

### 2.5. Statistics

Having 32 patients in each group (estimated point Ba improved 4.0 cm in the TVM alone group and 4.8 cm in the TVM + plication group) would provide the trial with 80% power, at a two-sided significance level of 0.05. Data are presented as the mean ± standard deviation (SD) or number/percent (n (%)). The Mann–Whitney U test or unpaired t test was used to compare two independent variables, and the Chi-square test was used to compare outcomes six and 12 months postoperatively. Fisher’s exact test was used to test categorical variables. Statistical analysis was performed using GraphPad Prism 6 (GraphPad Software, San Diego, CA, USA). *p* < 0.05 was considered statistically significant.

## 3. Results

**Figure 1:**
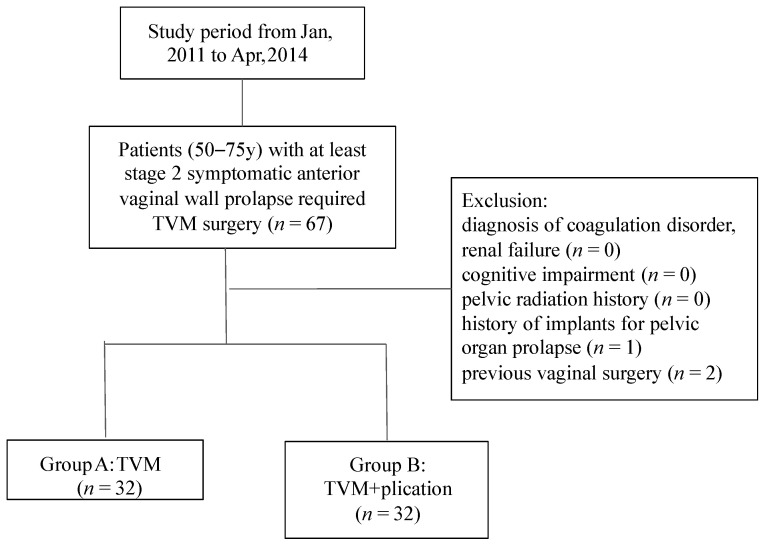
Study flow chart. TVM: transvaginal mesh.

From January 2011 to April 2014, 64 patients with anterior vaginal prolapse underwent anterior repair using the Avaulta Solo™ polypropylene mesh delivery system (Figure 1).

Group A included 32 patients who underwent TVM placement only, and group B consisted of 32 patients who received vaginal plication before TVM placement. There was no statistically significant difference in terms of the patient age, body mass index, parity, menopausal status, tobacco use, sexual activity, pre-/postoperative dyspareunia, and hysterectomy between the groups at baseline (Table 1). There were four and two dyspareunia noted preoperatively in groups A and B, respectively. After operation, the number of dyspareunia was increased to seven and six in groups A and B, respectively. However, there was no significant difference between the two groups (*p* = 0.1 in pre-op and *p* = 0.2 in post-op, Table 1).

Baseline POP-Q examinations were similar between the groups (Table 2). Postoperative POP-Q measurements at the 12-month follow-up were available in 30 and 31 patients from groups A and B, respectively. After a mean follow-up of 18.6 months (range 12–50), there were significant improvements at points Aa, Ba, C, Ap, and Bp in both groups (*p* < 0.001). In group A, the postoperative Aa and Ba measurements were more prolapsed compared to those in group B (−2.19 ± 1.22 vs. −2.53 ± 1.12) but did not achieve statistical significance (*p* = 0.26).

There was no significant difference in the concurrent procedures performed between the groups (Table 3).

According to a series of subjective questionnaire scores, both groups showed similar improvements in symptoms and quality of life (Table 4).

The primary outcome was to analyze the anatomic failure rate of anterior vaginal wall prolapse following a transvaginal polypropylene mesh with and without concomitant midline pubocervical fascia plication. Based on our definition of failure, the anatomic failure rate was higher in Group A (5/31, 16.1%) than in Group B for the 6- (12.5% vs. 0%) and 12-month follow-up (16.1% vs. 3.3%) periods, but it was not statistically significant (*p* = 0.19, Table 5).

Mesh exposure was detected in three of 61 patients (4.9%); two patients were from group A (6.7%), while one was from Group B (3.2%).

## 4. Discussion

The progressive application of the transvaginal permanent mesh in pelvic reconstructive surgery achieved outcomes superior to those of native tissue vaginal repair. It achieved a decreased awareness of prolapse and reoperation, but it was associated with a higher rate of complications, such as de novo stress urinary incontinence, iatrogenic bladder injury, or mesh exposure [15,22]. The utilization of transvaginal permanent mesh needs to be individualized, and the addition of concomitant fascial plication is based on the expertise of the physician. In our study, concomitant midline fascial plication yielded more durable results than TVM alone, probably because it provided a more robust structural support by directly repairing the defect. Moreover, concomitant midline fascial plication did not increase the mesh extrusion rate.

Regarding other plication techniques, a prospective study of 147 women who received a modified plication technique for cystocele repair and were followed-up for 82.4 months showed objective and subjective success rates of 89.8% and 92.2%, respectively [23]. Another study reported using a darned plication technique followed-up for one year and showed a satisfied outcome [24]. The previous study compared the use of biological graft with or without plication for cystocele repair [17]. They found that anatomic recurrence was higher in the nonplication group after 6 months of follow-up. The previous randomized study (132 patients recruited) compared biological graft with traditional anterior colporrhaphy (some patients with plication) for POP repair [25]. After a follow-up at three years, the recurrence rate in the two groups was the same. In our study, concomitant midline fascial plication provided a more durable anatomical support. However, there was no statistical significance.

During vaginal mesh surgery, concomitant midline fascial plication was not suggested due to the fact that it may be associated with mesh extrusion [19]. Risk factors for mesh exposure include concomitant hysterectomy, diabetes mellitus, and hypertension [26,27]. Nevertheless, these studies did not report midline fascial plication as a risk factor. Our study was the first study to demonstrate that concomitant midline fascial plication did not increase the mesh extrusion rate.

Maher et al. reported a Cochrane meta-analysis study that showed an overall 60% reduction in the risk of recurrent objective prolapse after TVM [15]. In our results, we defined surgical success based on objective and subjective outcome measures [28]. The success rates were comparable to the reported data range for other similar systems [19,29,30,31,32,33]. In our study, the group with the Avaulta Solo^TM^ device repair plus concomitant midline fascia plication had a 96.7 % success rate during the 12-month follow-up, while that of the TVM only group was 83.9%. This implied that concurrent plication provided additional anatomic support but did not enhance the outcome.

In a retrospective cohort study of TVM involving 120 women, Patrick et al. indicated that the Avaulta Solo device provided successful treatment for both anterior and posterior vaginal wall prolapses [19]. The mean follow-up time was 14.4 months. The surgical cure rate was 81% [19]. The incidence of de novo pain and mesh exposure was 3.3% and 11.7%, respectively [19]. Another study recruited 117 women with POP who received mesh (Avaulta and Pelvisoft) and conventional fascial plication techniques [34]. The anatomical failure rates after 2 years of follow-up were 19 and 27% in the mesh and conventional technique group, respectively [34]. Another study recruited 41 patients with POP repaired by Avaulta™ or Avaulta Plus™ and found that both devices were effective, safe, and improved life quality [35]. In our study, the improvements of the POP-Q parameters in the two groups were comparable.

According to a study by Barber et al., the within-treatment threshold of score difference is 45 points or more for the PFDI-20 questionnaire and 36 points or more for the PFIQ-7 to present clinical improvement [36]. In our study, the mean value difference between pre- and postoperation was appreciably smaller than the reference thresholds: 32.15 points for the PFDI-20 and 26.08 points for the PFIQ-7. These may be related to a milder symptom severity at baseline. All mean postoperative scores (along with their subscales) improved with statistical significance, except for the CRAIQ–7 score of group A (*p* = 0.26). Within the group compartment, similar postoperative subjective outcomes were observed, regardless of whether reinforcement with plication was done or not.

Although the transvaginal mesh achieved patient satisfaction and surgical correction outcome, about 5% of the patient samples experienced mesh extrusion. The US Food and Drug Administration (FDA) reclassified transvaginal mesh for prolapse repair to a class III (high risk) device in 2016 and required postmarket surveillance studies. In April 2019, the FDA ordered manufacturers to stop marketing transvaginal mesh kits for the repair of anterior/apical compartment prolapse. However, the use of SUI or transabdominal mesh for POP was still allowed. Annual check-ups are needed in women who received transvaginal mesh for the surgical repair of prolapse, and the benefit-risk profile of transvaginal mesh should be reviewed as more evidence becomes available.

Currently, whether TVM increases dyspareunia is controversial. A previous study showed an increasing odds ratio for postoperative dyspareunia (4.7, 95% CI 1.7–12.8) [37]. However, in another study, only one patient experienced dyspareunia (1.9%) in the mesh group [22]. The previous study using TVM surgery also showed an improvement of dyspareunia [38]. The previous systematic review showed that the percentage of dyspareunia ranged from 2.7% to 5.5% [39]. In our study, the number of dyspareunia was increased after operation in both groups but without statistical significance (4/10 to 7/10 in group A (*p* = 0.3), 2/14 to 6/14 in group B (*p* = 0.2)) and also without a significant difference between the two groups regarding pre-op and post-op dyspareunia (Table 1). The cause of the high percentage of dyspareunia may be due to the fact that we only counted the number of dyspareunia in sexually active women. The status of sexually active women in other studies is unknown.

The strengths of this study include those associated with prospective projects, its randomized design, and the 96.8% follow-up rate at 12 months. Moreover, a standardized surgical technique was performed by one surgeon throughout the study. The weaknesses of our study include a small sample size and the product availability. Lack of a previous power analysis and the small sample dimension were also noted.

In conclusion, the outcome (anatomical failure rate, subjective score, and mesh exposure rate) of TVM with concomitant midline fascia plication for anterior vaginal prolapse is comparable to that of TVM alone. More case numbers and longer follow-up times are needed to confirm our findings.

## Figures and Tables

**Table 1:** Patient demographics.

Patient Demographic	Group A (n = 32)	Group B (n = 32)	*p* Value
Age ^a^	63.7 ± 9.9	62.9 ± 10.1	0.7
BMI ^a^	25.4 ± 3.7	25.4 ± 2.9	0.9
Vaginal parity ^b^	3 (2–6)	3 (2–6)	0.8
Postmenopausal ^c^	26 (81)	30 (94)	0.2
Tobacco use ^c^	0 (0)	0 (0)	
Sexually active ^c^	10 (31)	14 (44)	0.4
Pre-op Dyspareunia ^c^ in sexually active	4 (40)	2 (14.3)	0.1
Post-op Dyspareunia ^c^ in sexually active	7 (70)	6 (42.8)	0.2
*p* for post-op compare to pre-op ^c^	0.3	0.2	
Previous surgery			
Hysterectomy ^c^	2 (6)	2 (6)	1.0

^a^ Data are mean ± standard deviation, unpaired *t*-test, ^b^ Data are median and range, Mann-Whitney test, ^c^ n (%), Fisher’s exact test, BMI: body mass index.

**Table 2:** POP-Q examination at baseline and postoperative points.

				POP-Q Measurements (cm)					
	Aa		*p*	Ba		*p*	C		*p*
	Group A	Group B		Group A	Group B		Group A	Group B	
Preoperative	2.50 ± 0.86	2.27 ± 0.98	0.3	3.30 ± 1.78	3.16 ± 1.87	0.7	1.48 ± 4.04	0.89 ± 3.66	0.5
Postoperative	−2.53 ± 1.12	−2.19 ± 1.22	0.2	−2.53 ± 1.12	−2.19 ± 1.22	0.2	−5.84 ± 7.40	−4.57 ± 2.18	0.3
	D		*p*	Ap		*p*	Bp		*p*
	Group A	Group B		Group A	Group B		Group A	Group B	
Preoperative	−3.07 ± 2.74	−3.13 ± 2.71	0.9	−0.91 ± 2.03	−1.09 ± 1.91	0.7	−0.81 ± 2.91	−0.50 ± 3.13	0.6
Postoperative	−4.85 ± 3.24	−5.22 ± 3.23	0.7	−2.76 ± 0.38	−2.73 ± 1.10	0.9	−2.79 ± 0.33	−2.77 ± 1.00	0.9
	gh		*p*	pb		*p*	tvl		*p*
	Group A	Group B		Group A	Group B		Group A	Group B	
Preoperative	3.88 ± 0.74	3.94 ± 0.70	0.7	2.22 ± 0.69	2.33 ± 0.66	0.5	7.23 ± 0.79	7.36 ± 0.61	0.4
Postoperative	3.16 ± 0.49	2.87 ± 1.16	0.2	2.87 ± 0.36	2.75 ± 1.10	0.5	6.68 ± 0.81	6.48 ± 2.54	0.6

Data are mean ± SD, unpaired *t*-test, Aa: the position of anterior vaginal wall 3 cm distance to hymen, Ba: the most prolapse region of anterior vaginal wall distance to hymen, C: cervix, D: posterior fornix, Ap: the position of posterior vaginal wall 3 cm distance to hymen, Bp: the most prolapse region of posterior vaginal wall distance to hymen, tvl: total vaginal length, gh: genital hiatus, pb: perineal body. Positive value: distance up from hymen (cm). Negative value: distance below the hymen (cm).

**Table 3:** Concurrent procedures.

Concurrent Procedures n (%)	Group A (n = 32)	Group B (n = 32)	*p* Value
VTH	16 (50)	12 (37.5)	0.4
Sling	12 (37.5)	18 (56.2)	0.2
Posterior Avulta	14 (43)	16 (50)	0.8
SSS	14 (43)	8 (25)	0.1
Posterior repair	28 (87.5)	28 (87.5)	1.0

Data was presented as n (%), Fisher’s exact test, VTH: vaginal total hysterectomy, SSS: sacrospinous ligament suspension.

**Table 4:** Subjective questionnaire scores at baseline and postoperative points.

				Subjective Questionnaire Scores					
Questionnaire	PFDI-20		*p*	POPDI-6		*p*	CRADI-8		*p*
	Group A	Group B		Group A	Group B		Group A	Group B	
Preoperative	40.75 ± 20.91	37.43 ± 16.47	0.4	20.05 ± 12.45	19.79 ± 10.36	0.9	3.91 ± 5.49	1.76 ± 3.45	0.01
Postoperative	5.92 ± 5.81	7.36 ± 7.03	0.3	1.43 ± 2.9	1.96 ± 3.17	0.5	0.59 ± 1.47	0.59 ± 1.47	1
	UDI-6		*p*	PFIQ-7		*p*	POPIQ-7		*p*
	Group A	Group B		Group A	Group B		Group A	Group B	
Preoperative	16.80 ± 9.07	15.89 ± 7.95	0.6	25.60 ±17.52	27.98 ± 15.43	0.7	17.26 ±11.39	17.11 ± 10.81	0.9
Postoperative	3.91 ± 3.81	4.82 ± 4.98	0.4	0	1.64 ± 4.62	0.05	0	0.15 ± 0.84	0.3
	CRAIQ-7		*p*	UIQ-7		*p*			
	Group A	Group B		Group A	Group B				
Preoperative	0.89 ± 2.24	1.04 ± 5.09	0.8	7.44 ± 9.67	9.82 ± 10.75	0.3			
Postoperative	0	0	0	0	1.49 ± 4.60	0.1			

Subjective questionnaire scores, Data are mean ± SD, unpaired *t*-test; *PFDI-20*, Pelvic Floor Distress Inventory, Short Form 20; *POPDI-6*, Pelvic Organ Prolapse Distress Inventory-6; *CRADI-8*, Colorectal-anal Distress Inventory-8; *UDI-6*, Urogenital Distress Inventory-6; *PFIQ-7*, Pelvic Floor Impact Questionnaire, Short Form 7; *POPIQ-7*, Pelvic Organ Prolapse Impact Questionnaire-7; *CRAIQ-7*, Colorectal-anal Impact Questionnaire-7; *UIQ-7*, Urinary Impact Questionnaire-7.

**Table 5:** The anatomic failure rate at the 6-month and 12-month follow-ups.

	6 Months Post Operation			12 Months Post Operation		
	N	n (%)	*p* *	N	n (%)	*p* *
Group A	32	4 (12.5)	0.4	31	5 (16.1)	0.19
Group B	31	0 (0)		30	1 (3.3)	
total	63	4 (6.3)		61	6 (9.8)	

*, Fisher’s exact test.

## Data Availability

The original data can be asked for, upon reasonable request, by contacting the corresponding author.

## Abbreviations

POP	pelvic organ prolapse
TVM	transvaginal mesh
POP-Q	pelvic organ prolapse-quantification system;
BMI	body mass index
PFDI-20	Pelvic Floor Distress Inventory, Short Form 20
POPDI-6	Pelvic Organ Prolapse Distress Inventory-6
CRADI-8	Colorectal-anal Distress Inventory-8
UDI-6	Urogenital Distress Inventory-6
PFIQ-7	Pelvic Floor Impact Questionnaire, Short Form 7
POPIQ-7	Pelvic Organ Prolapse Impact Questionnaire-7
CRAIQ-7	Colorectal-anal Impact Questionnaire-7
UIQ-7	Urinary Impact Questionnaire-7
SD	standard deviation
VTH	vaginal total hysterectomy
SSS	sacrospinous ligament suspension
TVL	total vaginal length
Gh	genital hiatus
Pb	perineal body
